# Rare cutaneous coelomycete infection in an immunosuppressed patient: When morphology is not enough

**DOI:** 10.1016/j.jdcr.2025.11.024

**Published:** 2025-11-26

**Authors:** Grace S. Saglimbeni, Maria Epino, A. Brian Mochon, Daniela C. Russi, Harper Price, Danielle Vargas de Stefano

**Affiliations:** a Creighton University School of Medicine, Phoenix, Arizona; b Division of Pathology and Laboratory Medicine, Phoenix Children's Hospital, Phoenix, Arizona; c Departments of Pathology and Child Health, College of Medicine-Phoenix, University of Arizona, Phoenix, Arizona; d Division of Dermatology, Phoenix Children's Hospital, Phoenix, Arizona

**Keywords:** cutaneous infection, immunosuppression, *Microsphaeropsis arundinis*, *Paraconiothyrium cyclothyrioides*

## Introduction

Coelomycetes are an artificial taxonomic group of filamentous fungi defined by phenotypic rather than genetic characteristics, producing conidia within fruiting bodies.^1^ Once considered environmental saprophytes, several coelomycetes, including the dermatologic pathogen *Neoscytalidium*,^2^ are now recognized as rare opportunistic human pathogens.^3^^,^^4^ Within this group, *Microsphaeropsis arundinis* and *Paraconiothyrium cyclothyrioides* are distinct species that exhibit similar clinical and morphologic features, making species-level distinction challenging even with molecular diagnostic methods.^2^^,^^5^

Human infections with coelomycetous fungi typically result from traumatic implantation, such as minor abrasions or gardening injuries, with exposure to contaminated soil.^4^^,^^5^ Because of their saprophytic origin and nondistinct morphology, infections by *M arundinis/P cyclothyrioides* are frequently misdiagnosed,^6^^,^^7^ delaying appropriate antifungal therapy.

Given these diagnostic and therapeutic challenges, infections with *M arundinis/P cyclothyrioides* require high clinical suspicion in immunosuppressed patients with nonhealing wounds. This case underscores the need for improved fungal identification tools and targeted treatment protocols.

## Case report

A 22-year-old man with hypoplastic left heart syndrome, postheart transplant (2017), Fontan-associated liver disease, and malnutrition from protein-losing enteropathy was admitted in July 2024 for protein-losing enteropathy management. Shortly after admission, he experienced right lower limb cellulitis, which he attributed to a superficial scrape sustained while playing soccer. Initially presumed bacterial, the infection was treated with intravenous vancomycin (500 mg every 6 hours for 10 days) and mupirocin ointment twice daily for 5 days. Despite this, symptoms worsened, with increased pain, marked erythema, and 3+ pitting edema of the lower portion of the leg.

**Fig 1:**
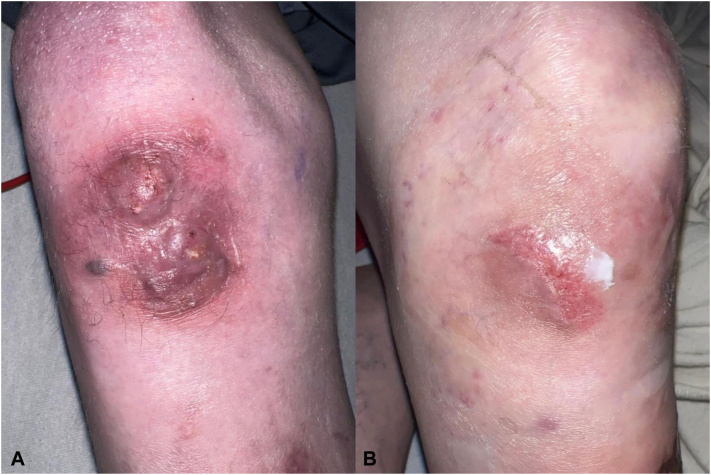
**(A)** On initial evaluation, the patient presented with a 3 × 3 cm erythematous, violaceous, and nodular plaque on the right knee that exhibited intermittent purulent drainage. **(B)** Clinical appearance after 5 months of posaconazole treatment. The lesion regressed, with scar formation and no signs of active infection.

The patient exhibited signs of shock, requiring intensive care. Blood cultures were negative. Although systemic symptoms stabilized with supportive care, the knee lesion progressed to a tender erythematous-violaceous plaque with erosion and intermittent purulent drainage (Fig 1, *A*).

**Fig 2:**
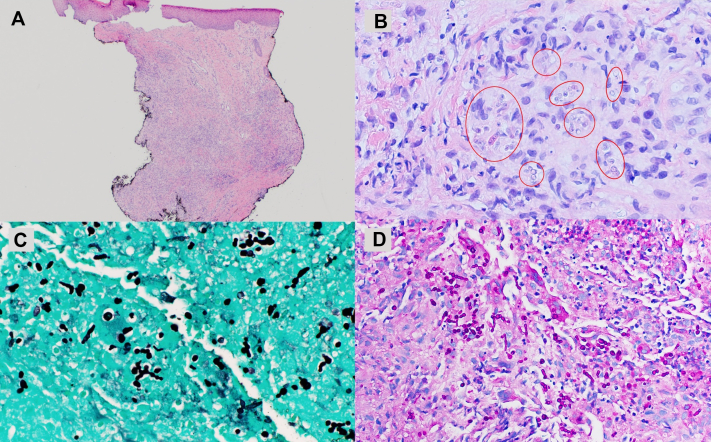
**(A)** Punch biopsy of right knee shows diffuse inflammatory process sparing epidermis and papillary dermis (hematoxylin and eosin stain, 1.25×). **(B)** At medium power, note the presence of a mixed chronic granulomatous inflammatory infiltrate with histiocytes, lymphocytes, and neutrophils. Microorganisms, possibly fungal forms, are visible on hematoxylin and eosin and indicated by circles (hematoxylin and eosin stain, 10×). **(C)** Grocott methenamine silver stain and **(D)** periodic acid–Schiff stain both highlight numerous septate hyphae and budding yeast forms within the dermis (Grocott methenamine silver stain, 60× oil; periodic acid–Schiff stain, 60× oil).

Infectious disease and dermatology consultations prompted wound culture and skin biopsy. Given the Arizona location, differential diagnoses included atypical mycobacteria, blastomycosis, sporotrichosis, cutaneous leishmaniasis, and other deep fungal infections. Culture morphology was consistent with a dematiaceous mold. Histologic examination revealed a flattened epidermis with underlying dermal scarring and acute and chronic granulomatous inflammation composed of histiocytic aggregates, lymphocytes, and neutrophils (Fig 2, *A*). Cluster of differentiation 1a immunostaining was negative for leishmaniasis, and acid-fast bacilli and Gram stains were negative for acid-fast bacilli and bacteria, respectively. Hematoxylin and eosin sections revealed abundant hyphae and budding yeasts lacking distinct morphologic features or pigmentation (Fig 2, *B*), prompting Grocott methenamine silver and periodic acid–Schiff stains, which confirmed the presence of fungal elements (Fig 2, *C*, *D*). Fontana–Masson was negative for pigment in tissue, supporting classification as hyalohyphomycosis.

The patient was started on intravenous liposomal amphotericin B (150 mg), but the lesion remained unchanged after several days. Matrix-assisted laser desorption/ionization time-of-flight on culture and 16S ribosomal DNA analysis with reflex next-generation sequencing on tissue identified coelomycetous fungi related to *M arundinis* or *P cyclothyrioides*. Following susceptibility testing, treatment was changed to oral posaconazole.

The lesion gradually regressed and showed marked improvement (Fig 1, *B*), with no fluctuance, drainage, induration, or new lesions. Prolonged antifungal therapy was advised.

## Discussion

Cutaneous *M arundinis/P cyclothyrioides* infections are exceedingly rare and often misdiagnosed as bacterial cellulitis, squamous cell carcinoma, or other chronic dermatoses.^3^^,^^5^ Reported cases predominantly occur in immunocompromised patients, particularly those receiving corticosteroid therapy, solid organ transplant recipients, and patients with chronic illnesses such as diabetes and autoimmune diseases.^1^^,^^4^^,^^5^^,^^7^ Infections commonly follow traumatic implantation, with plaques and nodules appearing on the hands, legs, or feet.^4^^,^^5^ In our case, the lesion developed from a wound contaminated with soil. Initial misdiagnosis led to a prolonged course of ineffective antibiotic treatment, highlighting diagnostic challenges of rare fungal infections.

In reported cases of *M arundinis/P cyclothyrioides* infections, histopathologic findings often include granulomatous inflammation with multinucleated giant cells, epithelioid histiocytes, neutrophils, and lymphocytes in the dermis.^1^^,^^6^^,^^8^ Fungal cultures typically show mold growth within 1 to 3 weeks, with colonies that darken with age and produce chains of smaller yeast-like cells and septate hyphae with swollen segments and distinguishable flask-shaped conidiomata containing elongated conidia.^5^^,^^9^ Our case revealed a similar granulomatous inflammation, along with septate hyphae and budding yeast. Although coelomycetous fungi are typically classified as phaeohyphomycotic given their ability to pigment in both culture and tissue, our case demonstrated pigmentation only in culture, and Fontana–Masson staining was negative for melanin, confirming classification as hyalohyphomycosis.

Because of the nonspecific morphology of the fungal structures, definitive identification, as in prior reports, required advanced techniques.^5^^,^^6^^,^^8^^,^^9^ Sequencing and matrix-assisted laser desorption/ionization time-of-flight narrowed the organism to *M arundinis* or *P cyclothyrioides*, but close genetic similarity prevented species-level distinction; therefore, they are addressed collectively. Prior studies demonstrate that internal transcribed spacer sequencing alone is often insufficient to differentiate these species due to high sequence homology. Supplementary sequencing of β-tubulin or actin genes is typically required for definitive identification.^10^ However, this level of sequencing is rarely available in routine clinical laboratories. Given their similar antifungal susceptibilities and treatment responses, distinguishing between the 2 may be of limited clinical significance.

The complexities of *M arundinis/P cyclothyrioides* infections are further compounded by their variable antifungal susceptibility across antifungal agents. In our patient, no improvement was noted with amphotericin B, whereas clinical response was achieved following transition to posaconazole. Previous reports have shown that *M arundinis/P cyclothyrioides* are responsive to posaconazole and terbinafine, but more resistant to itraconazole and voriconazole.^2^^,^^3^^,^^6^^,^^8^^,^^10^ Prolonged antifungal therapy is recommended to prevent recurrence and achieve complete resolution.

Regarding additional laboratory testing, matrix-assisted laser desorption/ionization time-of-flight offers faster results but is limited to material obtained from fungal culture, which typically requires weeks to grow. In contrast, 16S ribosomal DNA analysis with reflex next-generation sequencing can be performed on culture material or paraffin-embedded biopsy tissue. Although its processing time is longer, tissue preparation for histopathology is significantly faster than fungal culture, resulting in comparable overall turnaround times. In our case, the 16S ribosomal DNA analysis with reflex next-generation sequencing results were received first.

## Conclusion

This case underscores the importance of considering rare fungal pathogens, including *M arundinis/P cyclothyrioides*, in chronic, nonhealing wounds of immunosuppressed patients. Increased awareness and early fungal-specific diagnostics are crucial to improving outcomes and preventing misdiagnosis. Further studies on antifungal susceptibility and long-term management are warranted to optimize care for future patients facing similar infections.

## Conflicts of interest

None disclosed.
